# Rhenium-188 Labeled Radiopharmaceuticals: Current Clinical Applications in Oncology and Promising Perspectives

**DOI:** 10.3389/fmed.2019.00132

**Published:** 2019-06-14

**Authors:** Nicolas Lepareur, Franck Lacœuille, Christelle Bouvry, François Hindré, Emmanuel Garcion, Michel Chérel, Nicolas Noiret, Etienne Garin, F. F. Russ Knapp

**Affiliations:** ^1^Comprehensive Cancer Center Eugène Marquis Rennes, France; ^2^Univ Rennes Inra, Inserm, Institut NUMECAN (Nutrition, Métabolismes et Cancer)—UMR_A 1341, UMR_S 1241, Rennes, France; ^3^Angers University Hospital Angers, France; ^4^Univ Angers Univ Nantes, Inserm, CNRS, CRCINA (Centre de Recherche en Cancérologie et Immunologie Nantes—Angers)—UMR 1232, ERL 6001, Nantes, France; ^5^Univ Rennes CNRS, ISCR (Institut des Sciences Chimiques de Rennes)—UMR 6226, Rennes, France; ^6^Univ Angers PRIMEX (Plateforme de Radiobiologie et d'Imagerie EXperimentale), Angers, France; ^7^ICO (Institut de Cancérologie de l'Ouest) Comprehensive Cancer Center René Gauducheau, Saint-Herblain, France; ^8^ENSCR (Ecole Nationale Supérieure de Chimie de Rennes) Rennes, France; ^9^Emeritus Medical Radioisotopes Program, ORNL (Oak Ridge National Laboratory), Oak Ridge, TN, United States

**Keywords:** bone pain palliation, oncology, peptides, radioembolization, radionuclide therapy, radiopharmaceuticals, Rhenium-188

## Abstract

Rhenium-188 (^188^Re) is a high energy beta-emitting radioisotope with a short 16.9 h physical half-life, which has been shown to be a very attractive candidate for use in therapeutic nuclear medicine. The high beta emission has an average energy of 784 keV and a maximum energy of 2.12 MeV, sufficient to penetrate and destroy targeted abnormal tissues. In addition, the low-abundant gamma emission of 155 keV (15%) is efficient for imaging and for dosimetric calculations. These key characteristics identify ^188^Re as an important therapeutic radioisotope for routine clinical use. Moreover, the highly reproducible on-demand availability of ^188^Re from the ^188^W/^188^Re generator system is an important feature and permits installation in hospital-based or central radiopharmacies for cost-effective availability of no-carrier-added (NCA) ^188^Re. Rhenium-188 and technetium-99 m exhibit similar chemical properties and represent a “theranostic pair.” Thus, preparation and targeting of ^188^Re agents for therapy is similar to imaging agents prepared with ^99m^Tc, the most commonly used diagnostic radionuclide. Over the last three decades, radiopharmaceuticals based on ^188^Re-labeled small molecules, including peptides, antibodies, Lipiodol and particulates have been reported. The successful application of these ^188^Re-labeled therapeutic radiopharmaceuticals has been reported in multiple early phase clinical trials for the management of various primary tumors, bone metastasis, rheumatoid arthritis, and endocoronary interventions. This article reviews the use of ^188^Re-radiopharmaceuticals which have been investigated in patients for cancer treatment, demonstrating that ^188^Re represents a cost effective alternative for routine clinical use in comparison to more expensive and/or less readily available therapeutic radioisotopes.

## Introduction

During the last decades, new radionuclide-based targeted therapies have arisen as efficient tools for cancer and inflammatory lesions treatment. They are based on the use of unsealed radioactive sources emitting β^−^ or α particles, or Auger or low energy conversion electrons and aim at delivering tumoricidal ionizing radiation to tumor cells, while sparing healthy tissues (1–8). Several therapeutic radionuclides, essentially β^−^ emitters, are routinely used in clinics or actively investigated in clinical trials. Some of them are summarized in Table 1. Among them, ^188^Re is particularly attractive, thanks to its ideal properties [t_1/2_ = 16.9 h, E_βmax_ = 2.12 MeV, E_γ_ = 155 keV (15%)] and its on-demand availability at high-specific activity through its generator mode of production.

**Table 1:** Characteristics of important β^−^ emitters studied for radionuclide therapy.

**Radionuclide**	**t_**1/2**_ (days)**	**E_**β**_ (MeV) (%)**	**E_**γ**_ (keV) (%)**	**Tissue penetration range (mm)**	**Production method**
^32^P	14.3	1.71 (100)	/	8.7	Nuclear reactor
^47^Sc	3.4	0.600 (32)	159.4 (68.3)	3	Nuclear reactor Cyclotron
^67^Cu	2.6	0.575 (20)	184.6 (49.6) 93.3 (3) 91.3 (7.6)	2.2	Nuclear reactor Cyclotron
^89^Sr	50.5	1.492 (100)	/	8	Nuclear reactor
^90^Y	2.7	2.284 (100)	/	12	^90^Sr/^90^Y generator Nuclear reactor for microspheres labeling
^131^I	8	0.81 (90)	0.364 (81)	2.4	Nuclear reactor
^153^Sm	1.95	0.808 (21)	103 (28.3)	3	Nuclear reactor
^161^Tb	6.9	0.593 (100)	74.6 (10.2)	3	Nuclear reactor
^166^Ho	1.1	1.84 (50.5)	81 (6.4)	8.7	Nuclear reactor
^177^Lu	6.7	0.497 (79)	208 (11) 113 (6.4)	2.2	Nuclear reactor
^186^Re	3.8	1.07 (72)	137 (9)	4.5	Nuclear reactor
^**188**^**Re**	**0.7**	**2.118 (72)**	**155 (15)**	**11**	^**188**^**W/**^**188**^**Re generator Nuclear reactor**

*t_1/2_ (days), radioisotope half-life in days; E_β_ (MeV) (%), maximum particle energy and respective decay abundance shown in parentheses; E_γ_ (KeV) (%), gamma ray energy useful for imaging and respective abundance in total energy emission shown in parentheses; Tissue penetration range (mm), maximum tissue penetration shown in millimeters. Bold values indicates 188Re*.

**Figure 1:**
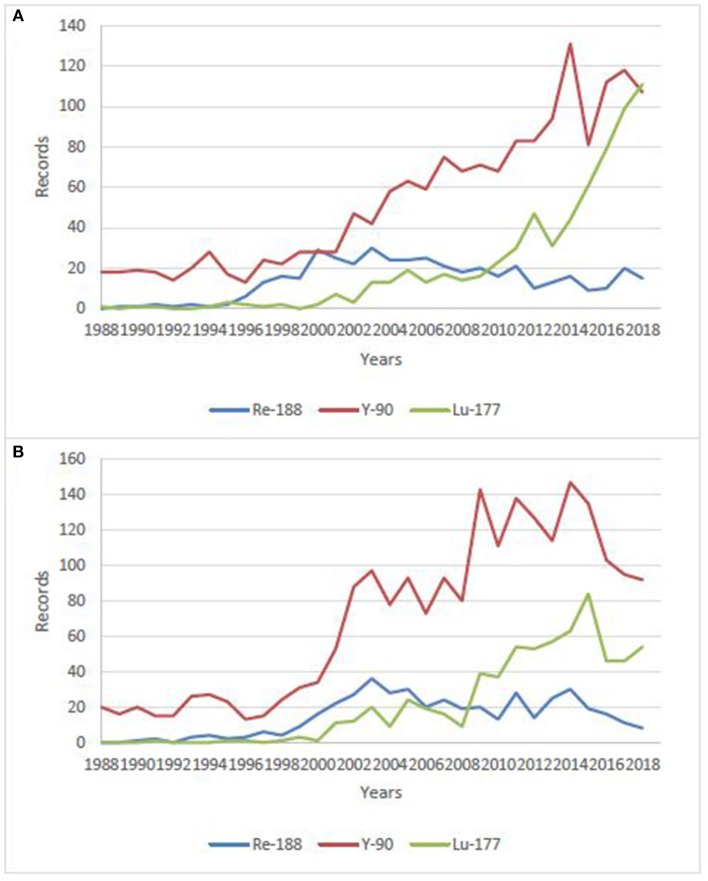
Number of publications/year on clinical use of ^188^Re, ^177^Lu and, ^90^Y over the last 30 years. **(A)** data from Sci-Finder, ^©^2019 American Chemical Society, **(B)** data from Web of Science, ^©^2019 Clarivate Analytics.

Rhenium is the 3rd-row congener of transition metal elements in Group VIIB, after manganese and technetium, which, with its isotope technetium-99 m (t_1/2_ = 6 h, E_γ_ = 141 keV), has been the workhorse of nuclear medicine for more than half a century (9–11). It has a rich chemistry, with oxidation states ranging from −1 to +7 and coordination numbers up to nine. Rhenium is able to complex with a variety of ligands and bifunctional chelating agents (12–16). It possesses two potentially useful therapeutic isotopes, ^186^Re and ^188^Re (1). Both can be produced non-carrier-added (nca), but ^188^Re is produced with high specific activities, thanks to its generator mode of production, while ^186^Re is essentially reactor-produced with low specific activity, but research is currently conducted on cyclotron production of nca ^186^Re (17, 18). Likewise, both possess γ emissions which allow for imaging and dosimetry calculations. ^186^Re has a lower β^−^ emission with a maximum tissue penetration of 4.5 mm, which is more or less half that of ^188^Re (11 mm), making ^186^Re particular suitable for treating small to mid-sized tumors while ^188^Re is a better match for larger-sized tumors. Considering half-lives, ^188^Re has a relatively short one (17 h) which restricts its use to agents with rapid target uptake and non-target tissue clearance, while ^186^Re can also be employed in targeting agents with longer biological half-lives, like antibodies. Based on chemical similarities and the availability of non-radioactive isotopes—which is not the case for technetium—rhenium has been used as a surrogate for technetium-99 m to elucidate structures and mechanisms (19–21). On the other hand, ^99m^Tc-labeled radiopharmaceuticals likewise serve as a model to prepare ^186/188^Re-radiotracers using similar labeling methods (22, 23). However, despite close properties, there are notable differences in the reactivity of technetium and rhenium, particularly concerning their reaction kinetics and redox behaviors (24, 25). Perrhenate is much more difficult to reduce than pertechnetate, which is of prime importance, since this is the form obtained from the generators. This rich but difficult chemistry—which has been thoroughly reviewed recently and do not enter the scope of this review (26), coupled with the current limited availability of pharmaceutical-grade rhenium-188, may explain why ^188^Re-radiopharmaceuticals have not yet gained wide acceptance, while the use of more convenient therapeutic isotopes (simple, straightforward chemistry, and high production capacities), such as ^90^Y and ^177^Lu, is steadily increasing. This is clearly visible when making a bibliographical search on these isotopes, combined with “clinical” research term (Figure 1), despite the expected considerably higher costs. There are nonetheless research groups actively working on ^188^Re-labeled compounds all over the world, aiming at demonstrating the potential clinical usefulness of ^188^Re-radiopharmaceuticals for the treatment of various benign and malignant conditions. ^188^Re, under different forms, from small labeled molecules to large antibodies, or loaded into particles, from nanosized colloids to microspheres, has been investigated in various malignant diseases. Several clinical trials are currently going in progress, and some very promising new compounds are in advanced preclinical evaluation and deserve further investigation in patients.

## ^188^Re Production

**Figure 2:**
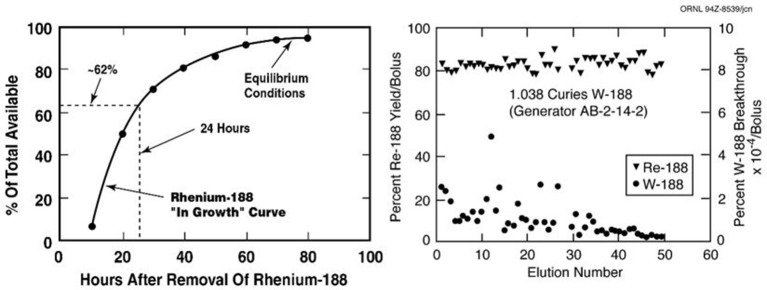
The useful shelf-life of the ORNL alumina-based ^188^W/^188^Re generator shows consistently high ^188^Re-perrhenate yields and low ^188^W breakthrough over at least 2 months (Image property of ORNL, courtesy of Dr. Russ Knapp, Oak Ridge, TN).

The attractive performance properties of the alumina-based ^188^W/^188^Re generator system have been widely described (27–32). However, factors which will affect the hopeful broader use of ^188^Re in routine clinical practice include the costs and required routine reactor production of sufficient activity levels of ^188^W. These are key issues which have challenged the broader use and routine clinical introduction of ^188^Re-labeled radiopharmaceuticals. One very attractive characteristic for routine clinical use of the ^188^W/^188^Re generator is the relatively rapid ^188^Re daughter in-growth (~60% in 24 h) following bolus elution, which means the generator can be used on a daily basis to optimize clinical use of ^188^Re-labeled therapeutic agents (Figure 2). The many advantages for radiotherapy with ^188^Re would be expected to maintain broad interest in the continued availability of the ^188^W/^188^Re generator system. Unfortunately, efficient generator utilization has generally not been the case at most institutions evaluating the early stage clinical trial-based evaluation of ^188^Re therapeutic agents. The limited *ad ho*c use of the ^188^W/^188^Re at many institutions has been often particularly inefficient, because of relatively high generator costs, discussed below. To offset these high costs, one strategy for the most cost-effective generator use, is installation of the generator at a central radiopharmacy site located in a high-density patient population area, where unit ^188^Re doses can be dispensed to surrounding clinics. Another strategy would be generator installation at specialized regional clinical centers where patients could be referred from the surrounding area. The cost-effective use of the ^188^W/^188^Re generator is particularly attractive for use in developing countries because of the low unit dose costs generator system is effectively used (33).

### Reactor Production of ^188^W

The reactor production of ^188^W by double neutron capture of enriched ^186^W targets by the [^186^W(n,γ)^187^W(n,γ)^188^W] double neutron capture pathway has been demonstrated in some detail (34, 35). Naturally occurring stable tungsten isotopes are: ^182^W (26.5%), ^183^W (14.3%), ^184^W (20.64%), and ^186^W (28.43%). Since neutron capture will thus produce a variety of generally unwanted radioisotope products, isotopically enriched ^186^W (~ > 90%) is used for reactor production of ^188^W. Facilities in the U.S. (ORNL) and in the Russian Federation had traditionally enriched isotopes of high Z metallic elements such as ^186^W, and significant inventories of ^186^W are still available. However, the aging and expensive calutron enrichment facilities which had been operated at ORNL since the 1940's, were taken out of operation in 1998. Significant inventory levels of ^186^W are still available at ORNL, and the good news is that the ORNL isotope enrichment capability is now being re-established. A comprehensive detailed overview on the issues associated with reactor production of ^188^W was published by the International Atomic Energy Agency (IAEA) in 2010 (36). Although the availability and broad use of particle accelerators for the production of many medical radioisotopes would be expected to be considerably less expensive than reactor production, no methods have yet been described for the practical accelerator production of ^188^W.

The neutron cross sections (σ, probability of nuclear neutron capture) for ^188^W production from neutron irradiation of ^186^W have been well studied (37). Since the thermal neutron cross section values are a function of the square of the thermal flux for such a double neutron capture process, the ^188^W product yield, for instance, is essentially doubled by a two-fold increase in neutron flux. Thus, the thermal neutron flux is an important crucial issue for production of ^188^W. For this reason, high flux nuclear reactors with thermal neutron flux values of at least 10^14^ thermal neutrons/cm^2^ are generally felt to be required for effective ^188^W production (i.e., sufficient specific activity for generator use). The ^188^W yields at these thermal neutron flux values are about 5–10 mCi/mg ^186^W target, but depend on a variety of factors regarding the reactor used.

Important factors for reactor production which are beyond the scope of this discussion include the reactor neutron flux spectrum, thermal flux values, reactor cycle, target volume capabilities, shutdown between reactor cycles, etc. The saturation of ^188^W production and maximization of specific activity are important factors to optimize ^188^W production and processing costs. At the ORNL HFIR, for instance, two successive reactor cycles are optimal and practical for ^188^W saturation as a balance between specific activity increase and operation costs, since the down time between cycles is usually only 1 week. Another issue is the radioactive impurities which are produced as irradiation increase and which should be minimized. By many standards, these modest production activity yields and low specific activity may seem low, but in the case of the ^188^W/^188^Re generator, these factors are significantly and practically off-set by several attractive operational parameters (38). These factors include the long ^188^W 60-day physical half-life, the high routine daily ^188^Re generator elution yields of 60–80% and the very long useful ^188^W/^188^Re operational shelf-life of several months.

#### ^188^W Target Material, Irradiation, and Processing

Because reactor irradiation costs are usually based on the target volume, the early use of low density encapsulated ^186^W targets was replaced at some institutions by use of high density pressed/sintered ^186^W targets (39), which greatly increases the ^186^W mass within the target capsule, thus significantly decreasing the costs per Ci of the ^188^W produced. *Ergo*, more target mass allows production of higher product activity levels. For this reason, the ^188^W has been usually produced at the following three institutions (33): High Flux Isotope Reactor in Oak Ridge, TN *(1.8x10*^15^*/neutrons/cm/sec)*, the SM3 Reactor in Dimitrovgrad, Russian Federation *(3x10*^15^*/neutrons/cm/sec)*, and the BR2 reactor in Mol, Belgium *(1x10*^15^*/neutrons/cm/sec)* (40). Traditional processing of reactor-irradiated enriched ^186^W metal oxide powder targets involved caustic dissolution (41, 42). Processing of the preferred pressed ^186^W metal targets, involves initial high temperature conversion of the irradiated metallic ^188^W/^186^W (i.e., only low percent of ^186^W atoms are activated) with the oxygen in atmospheric air using a quartz glass reaction apparatus (39). Subsequent dissolution of the [^188^W]WO_2_ product with caustic provides the ^188^W-tungstate ([^188^W]Na_2_WO_4_) stock solution which is then acidified to tungstic acid ([^188^W]HWO_4_) on an on-required basis for generator fabrication.

#### ^188^W Target Material Recovery

Because only a small fraction of the enriched ^186^W target atoms are activated to ^188^W during the reactor irradiation, once the activity levels of eluted ^188^Re-perrhenate equilibrium from the generators reach activity levels which are too low and are impractical for radiopharmaceutical preparation, the non-activated ^186^W remaining on the generator matrix can be removed by basic elution and then reprocessed for subsequent activation (43).

### ^188^W/^188^Re Generator Fabrication and Use

#### Generator Fabrication

Similar to fabrication of the ^99^Mo/^99m^Tc generator, activated alumina is currently the most widely used absorbent for fabrication of the ^188^W/^188^Re generator column (44, 45). Significant R&D has been devoted over the last three decades to the development of ^188^W/^188^Re generator prototypes, most notably in studies supported by the IAEA. A variety of other methods have been evaluated for separation of ^188^Re from ^188^W, although detailed discussion of these strategies is beyond the scope of this overview and has been reviewed elsewhere (32). As a brief summary, in addition to the use of alumina, other metal oxides, such as zirconium and titanium tungstates, nanocrystalline titania, polymeric titanium oxychloride sorbets and hydroxyapatite, have also been evaluated, and alternative methods which have been investigated for separation of ^188^Re from ^188^W include solvent extraction and electrochemistry. Evidently, these methods have not progressed further since the alumina-based ^188^W/^188^Re adsorbent has been extensively evaluated in the clinical setting with excellent performance.

For the alumina-based generator, the processed basic sodium tungstate stock solution ([^188^W]Na_2_WO_4_) is then converted to tungstic acid by acidification with HCl to pH 2–3 and then slowly percolated through the saline-washed alumina column which is then washed thoroughly with additional saline solution.

#### ^188^W/^188^Re Generator Elution

The standard alumina-based generator is eluted with saline at a slow flow-rate of typically 1–2 mL/min., with the volume based on the size of the generator (i.e., “void volume”) to insure complete removal of the ^188^Re bolus. Some institutions have instituted the use of semi- or totally-automated elution systems (46–48). These methods have helped move use of the generator forward, and are important to insure reproducible results and reduce the user radiation burden. Microprocessor-controlled detector systems have also been often incorporated for selection of only the peak ^188^Re activity volume, in order to optimize the bolus ^188^Re volume. The potential importance for use of these methods is dependent on the particular clinical application and thus the total ^188^Re activity and specific activity requirements.

#### ^188^Re Eluent Concentration

Because of the relatively low specific activity of reactor-produced ^188^W (typically 5–10 Ci/g ^186^W), the mass of alumina to bind the tungstic acid solution ([^188^W]HWO_4_) must be sufficient for irreversible ^188^W-tungstic acid binding, typically 10 grams alumina/Ci of ^188^W. In contrast, because of the very high specific activity of fission-produced ^99^Mo, only very low amounts of alumina are required for the ^99^Mo/^99m^Tc generator system, resulting in very high specific volume of the saline bolus eluents (mCi/mL saline). Because of the much lower specific activity of ^188^W, higher volumes of saline are thus required for elution of ^188^Re eluents, resulting in relatively low specific volumes. With high activity (5–10 Ci) ^188^W/^188^Re generators, especially for initial use, bolus concentration is often unnecessary since the ^188^Re specific volume is adequate. However, use of bolus concentration is very important to extend generator shelf-life almost indefinitely and for use of generators fabricated with lower specific activity ^188^W.

Thus, a convenient and useful strategy for extending the ^188^W/^188^Re generator half-life involves post-elution concentration of the ^188^Re bolus solution. Generally, all methods which have been evaluated are based on a similar strategy, focused on the separation of the eluent anions for subsequent specific trapping of the eluted ^188^Re-perrhenate. The first and currently most widely used convenient method involves a simple two-column tandem flow-through system based on the specific separation of the macroscopic levels of the chloride anions (Cl^−^) from the saline eluting solution from the microscopic levels of the eluted perrhenate anions ([^188^Re]Re${\text{O}}_{4}^{-}$) (49, 50). The system, which was first described by Blower for concentrating ^99m^Tc generator eluates (51), is based on the specific trapping of the chloride anions on a silver-nitrate-based anion trapping column through which the perrhenate anions flow through and then are subsequently retained in a second anion trapping column. The perrhenate is then obtained by low volume elution of the second column, providing very high ^188^Re specific volume solutions. The increase in ^188^Re specific volume from elution of the initial of the generator column can be at least 8–10-fold. An effective similar system uses salts of weak acids such as ammonium acetate for generator elution with subsequent trapping of [^188^Re]-perrhenate (52). Subsequently, a variety of potentially useful alternative methods have also been described (53–57).

#### Availability of GMP/Pharmaceutical-Grade Generators

Of course, for both early stage through routine clinical applications of ^188^Re-labeled therapeutic radiopharmaceuticals, GMP-manufactured generators are required, with subsequent GMP preparation of specific therapeutic agents. One previously widely used ^188^W/^188^Re generator had been available for several years form the Oak Ridge National Laboratory (ORNL) in the U.S., which were manufactured and distributed throughout the world as a non-sterile GMP-generator. Over about a 20-year period, several hundreds of these generators had been use in both pre-clinical and for a variety of clinical applications. The GMP generators are no longer available from ORNL. More recently, IRE in Fleurus, Belgium, has begun routine production and distribution of the “Rheni Eo” ^188^W/^188^Re generator system equipped with a GMP remote-controlled bolus concentration system. Because the reactor-production/processing/cGMP costs are not insignificant, the radiopharmacy use of the generator system and use of the eluted ^188^Re must be optimized to amortize the initial generator investment costs. In many cases through the last decades, the radiopharmacy/clinical use of these generators had not been optimized, thus resulting in unacceptably high unit ^188^Re costs.

## ^188^Re-Labeled Small Molecules

[^188^Re]-perrhenate, due to its structural analogy with iodide (near ionic radii, identical charge), has been tested in models of cancers expressing the sodium/iodide transporter (NIS). NIS is a plasma membrane protein that mediates active iodide transport into the thyroid gland and several extra-thyroidal tissues, and notably breast cancer, which naturally expresses NIS in more than 80% of cases. Beside, NIS can be used both as a reporter and as a therapeutic gene, making it possible to image and treat the tumor with radioiodide (^131^I), just as in differentiated thyroid cancer (58, 59). Using ^188^Re instead of ^131^I seems to be a potential alternative (60), and has been investigated in NIS-expressing mammary tumors (61, 62), as well as prostate (63), liver (64) and cervical cancers (65), after NIS gene transfection with adenoviruses or lentiviruses. This use of a virally-directed radioisotope therapy, called radiovirotherapy, seems particularly attractive (66), but it needs to be demonstrated in patients.

Apart from this above example, to be able to deliver its therapeutic activity to the tumor cells, rhenium-188 needs to be attached to a tumor-seeking agent, either based on specific site affinity or a particular mechanism (67).

### ^188^Re-DMSA for Medullary Carcinoma

**Figure 3:**
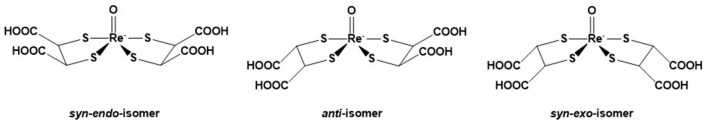
^188^Re-DMSA isomers.

DMSA (meso-2,3-dimercaptosuccinic acid) is a small molecule which exists in two forms labeled with technetium-99 m. Tc(III)-DMSA is a routinely used radiopharmaceutical useful for renal imaging, to evaluate renal structure and morphology, particularly in pediatric imaging for detection of scarring and pyelonephritis (68), while ^99m^Tc(V)-DMSA is useful for imaging medullary carcinoma of thyroid, head and neck tumors and metastasis from breast carcinoma to liver, brain and skeleton (69). It was thus logical that ^188^Re(V)-DMSA was envisaged to be useful for the treatment of the above cancers. Three isomers (syn-endo, syn-exo and anti) are formed (Figure 3). The isomeric composition may vary depending on the conditions of preparation. The complex is synthesized from the commercial kit for ^99m^Tc. Bolzati et al. have proposed a new approach (70) for the synthesis of ^188^Re(V)-DMSA, requiring less stringent conditions. The biological properties of ^188^Re(V)-DMSA have been studied in animals and humans (71, 72). The results in patients showed a selective attachment to tumor tissues, particularly to metastatic bone cancer originating from prostatic carcinoma, similar to that of the technetium analog (73). The limiting factor for the use of ^188^Re(V)-DMSA may be its high renal accumulation, higher than the ^99m^Tc-counterpart (74), though, according to Blower et al. (73), this potential kidney irradiation should not be precluding a therapeutic or palliative use of ^188^Re(V)-DMSA.

### Bone Pain Palliation Agents

Skeletal metastases occur in ~50% of women with breast cancer, the most common cancer in women, and in 80% of patients with prostate carcinoma, the second most common cancer in men, as well as some other tumors, such as myeloma or lung cancer (75). Medullary infiltration and matrix involvement are usually associated. Tumor infiltration is directly responsible for the pain phenomenon. Approximately half of the patients will continue to have substantial bone pain after the standard surgical and/or non-radiologic treatment options are exhausted. Metabolic radiotherapy offers a therapeutic alternative that is particularly noteworthy (76–78). All localizations are treated immediately by means of a single intravenous injection. Peptide receptor radionuclide therapy (PRRT) with somatostatin analogs (^177^Lu-octreotate) and PSMA ligands has also demonstrated its potential clinical usefulness for bone metastases arising from neuroendocrine tumors and metastatic castration-resistant prostate cancers (mCRPC), respectively, (79, 80). The idea of using therapeutic radioisotopes to treat the pain of bone metastases dates back to the 1940s. The first tests were due to Lawrence (81) who used phosphorus-32 as an orthophosphate. However, the major disadvantage of ^32^P is its high hematological toxicity related to the importance of the activity delivered to the bone marrow. For over 20 years, a wide variety of radiopharmaceuticals that can be used to deliver radiation to metastatic bone sites have been developed (82–87). Currently, four are commercially available: ^89^SrCl_2_ (Metastron®), ^223^RaCl_2_ (Xofigo®) ^153^Sm-EDTMP (Quadramet®), and ^186^Re-HEDP (^186^Re-etidronate®). ^89^Sr and ^223^Ra are used as such because of their natural tropism for bone, mimicking the Ca^2+^ cation, whereas ^153^Sm and ^186^Re are used as phosphonates (EDTMP = ethylenediaminetetramethylene phosphonate and HEDP = hydroxyethylidene diphosphonate), which are molecules having a very strong affinity toward calcium present in the actively growing bone. To date, ^223^RaCl_2_ is the only one with a proven benefit on overall survival (86, 88).

**Figure 4:**
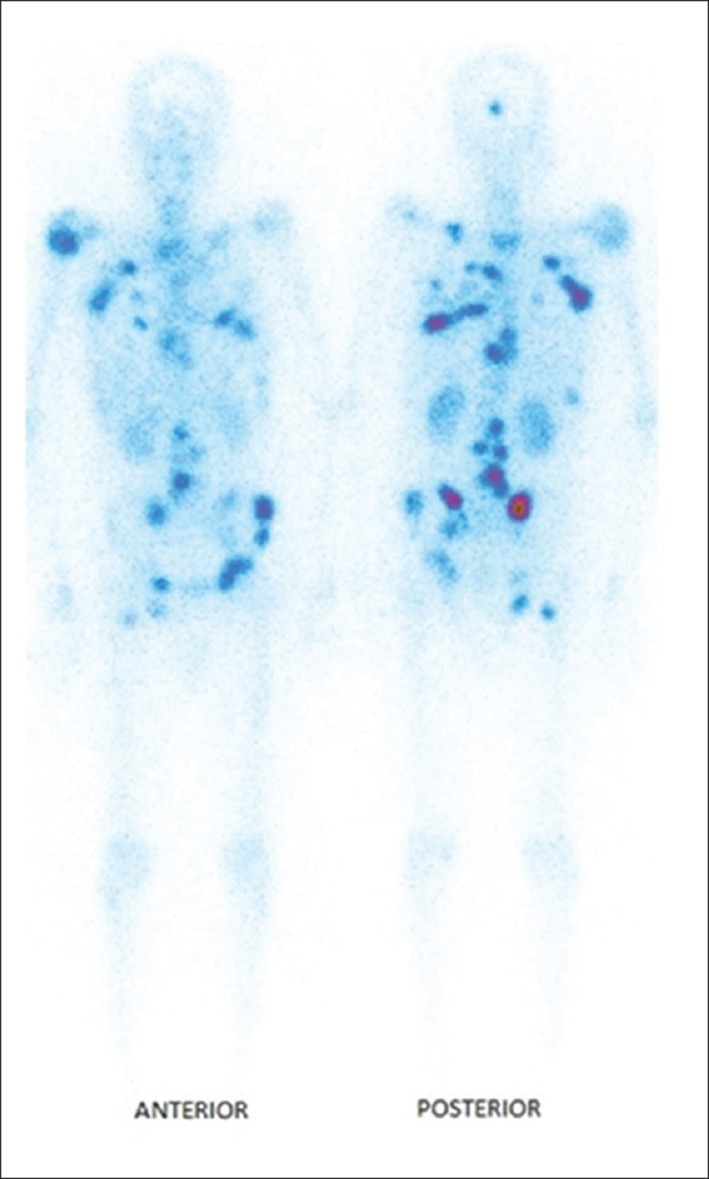
Typical distribution of ^188^Re-HEDP, 24 h post-injection [from Shinto et al. (105), available under the terms of the Creative Commons Attribution-NonCommercial-ShareAlike License (CC BY-NC-SA)].

In a recent review on new radionuclides for bone pain palliation, ^188^Re appears to be one of the most promising candidates (89). The first example of the use of ^188^Re-HEDP to treat patients was reported by Maxon et al. (90). The cost and availability of ^188^Re make it a radioisotope more interesting than ^186^Re. In addition, it is expected that the maximum tolerated dose by the patient is more important for ^188^Re than for ^186^Re (91) and the shorter life of ^188^Re allows to fractionate the injected doses (92–94). The comparison of the biodistribution of ^186^Re-HEDP and ^188^Re-HEDP showed an identical behavior for the two molecules (95, 96). ^188^Re-HEDP also demonstrated similar efficacy in comparative studies with ^186^Re-HEDP, ^153^Sm-EDTMP and ^89^SrCl_2_ (97, 98). A Phase III trial has recently started to compare its efficacy to ^223^RaCl_2_, in patients with castration-resistant prostate cancer metastatic to bone (RaRe trial, NCT03458559). The maximum tolerated dose (MTD) of ^188^Re-HEDP was established to be 3.3 GBq in a dose escalation study by Palmedo et al. (91). Two other dosimetry-based studies demonstrated treatment was safe with an acceptable radiation-absorbed dose to the normal bone-marrow and no limiting hematological toxicity (92, 99). In a study with 15 patients suffering from breast or prostate cancer bone metastases (100), Liepe et al. reported pain relief in 80% of the patients, with 20% patients who were pain-free and could discontinue their analgesics. The same team later reported similar results in 27 prostate cancer patients (101). In a study on patients with lung cancer bone metastases (102), 46% of the patients were able to suspend their analgesics intake. As can be seen, tolerance and efficacy are highly dependent on the primary tumor site. In a study with 61 patients with skeletal metastases from lung, prostate, breast, renal, rhinopharingeal and bladder cancers, pain reliefs were achieved for, respectively, 77, 80, 83, 50, 50, and 100% of the patients (103), while in another study with 64 patients with prostate, breast, lung and liver cancer (104), pain relief was reported for 84.62, 78.57, 62.50, and 55.56%, respectively. In a very recent study by Shinto et al. (105), overall response rate was 89.5% in 48 patients with metastases from different types of cancers. Results were not detailed according to the primary tumor (Figure 4). Lange et al. specifically studied the impact on quality of life, proving the routine clinical benefit of ^188^Re-HEDP therapy (106). A small study by Sabet et al. on 6 patients, failed to demonstrate the usefulness of salvage therapy with ^188^Re-HEDP for patients with progressive bone metastases after ^177^Lu-octreotate therapy (107). It has been demonstrated that combination with a radiosensitizer, like capecitabine or taxane, could prove useful and lead to increased efficacy (108, 109). There is also evidence that, compared to single injection, multiple injection could lead to improved overall survival (88, 93, 94). In their retrospective analysis, Biersack et al. reported overall survivals increasing with the number of injections (from 1 to 3), from 4.50 to 15.66 months. The ongoing RaRe trial should answer this question.

An important point in the preparation of ^188^Re-HEDP is the necessity of decreasing the specific activity by adding “cold” rhenium (*aka* carrier) in order to have good bone fixation. Several studies have investigated the influence of the reaction conditions and kit composition on final product's stability and *in vivo* behavior (110–118). All of them pointed out that the addition of carrier was crucial. A GMP grade kit for the preparation of ^188^Re-HEDP has recently been described (119) and a standard procedure following the ICH Q8 guideline, and investigating the critical step parameters, has been reported by the same team (118).

Another bisphosphonate has recently been investigated in patients (120). In a Phase I/II trial including 63 patients, ^188^Re-zoledronic acid was compared with ^89^SrCl_2_, and demonstrated similar safety profile. In terms of survival, it seems treatment was more beneficial to breast cancer patients than prostate cancer ones, although the difference was not significant. Several other bisphosphonates and aminophosphonates derivatives have been the subject of development, but have not reach the clinic yet (121). As noted above, ^188^Re(V)-DMSA exhibited a high affinity for bone metastases from prostate cancer, but no further study was ever carried out following the one by Blower et al. (73).

## ^188^Re-Labeled Peptides and Antibodies for Hematological and Solid Tumors

Tumor cells overexpress a large range of cellular receptors not or poorly expressed by normal tissues. It is, in consequence, possible to selectively target these receptors through the use of targeting moieties with high affinity and selectivity for these receptors. For instance, antibodies targeting antigens expressed on the surface of the tumor or peptides acting as agonist or antagonist to those receptors. Radioimmunotherapy (RIT) and peptide receptor radionuclide therapy (PRRT) have demonstrated their clinical effectiveness, with some radiopharmaceuticals currently approved and a many more under clinical investigation (122–126). Best responses to RIT have been obtained with hematopoietic malignancies, in contrast to solid tumors, in spite of the delivery of somewhat low doses. This can be explained by a better vascularization, more homogenous tumor cell population and the contribution of apoptotic and immune mechanisms (127).

### RIT With ^188^Re-Labeled Antibodies

Antibodies have long circulating times, so ^188^Re, with its short half-life, might not be the best suited radionuclide for antibody labeling, for which ^186^Re, with its 3.8-day half-life, could be more appropriate (Table 1). Nonetheless, several antibodies or antibody fragments have been labeled with ^188^Re, by direct or indirect methods (128), and investigated preclinically in a wide variety of tumors, like anti-CD52 and anti-CD66 in leukemia (129, 130), anti-CD20 (rituximab) in lymphoma (131), trastuzumab derivatives in breast, nasopharyngeal or prostate carcinomas (123–134), bevacizumab in non-small-cell lung cancer (135), cetuximab in lung cancer (136), anti-EGF-R antibody h-R3 (nimotuzumab) in glioma (137), anti-CEA MN-14 antibody in gastrointestinal cancers (138), C595 (anti-MUC1) in transitional cell bladder carcinoma (139), MEM238 (IGF2R-specific) in osteosarcoma (140), mAbCx-99 (anti-Ck19 antigen) and C1P5 (targeting E6 viral oncoprotein in human papillomavirus positive cervical cancers) in cervical cancers (141, 142), Listeria-binding antibodies in metastatic pancreatic cancer (143) or melanin-binding IgG or IgM in melanoma (144). Some of them have made their way to the clinics.

BW 250/183 [anti-CD66 (a, b, c, e) antibody], of murine origin and of IgG1 isotype, has a high affinity for the CD-66 antigen present on the cells of the granulocyte line. It is non-specifically directed against a surface glycoprotein, NCA-95, overexpressed on the membrane surface of human myelocytes and metamyelocytes. Radiolabeled with ^188^Re, it has been tested as an adjunct in marrow transplant packaging in 12 patients with acute leukemia (145) and in 36 patients with acute myeloid leukemia or myelodysplastic syndrome (146). Initial results suggest delivery of a significant radiation dose to bone marrow and minimal toxicity, demonstrating its potential clinical interest prior to bone marrow transplantation. Indeed, injection of radiolabeled antibodies maximizes immunosuppresion in the marrow while avoiding extra-medullary adverse effects (147). A phase I/II study was of particular interest in patients over 55 years of age with a high risk of acute leukemia (148, 149). Nevertheless, one of the main complications is the appearance of transplantation-related toxicity (150) and particularly nephropathies (151). To minimize its adverse effects, the use of ACE inhibitors, angiotensin receptor blockers or forced diuresis is recommended (152). A Phase II study demonstrated that combination of ^188^Re-radioimmunotherapy with reduced-intensity conditioning was feasible and effective (149), but that dose-reduction of alemtuzumab did not impact overall and disease-free survival (152). ^188^Re-RIT has also been investigated in patients with non-Hodgkin's lymphoma, using ^188^Re-rituximab (131). Preliminary dosimetric results indicate it could compare favorably with ^131^I-rituximab.

A study by Juweid et al. investigated the use of ^188^Re-labeled antibodies in solid tumors such as gastrointestinal or pancreatic cancer (138). They used an antibody of murine origin, MN 14, directed against the specific CEA epitope. Their results showed that the stability of the selected antibody was not the most suitable especially in patients with weak CEA expression and low tumor burden. The presence of a tumor that is too large and poorly vascularized decreases the therapeutic efficacy given the slow biodistribution of the antibodies. The authors proposed to develop more stable compounds *in vivo* using multi-step delivery system, to use bivalent antibodies or antibody fragments. However, the use of antibody fragments could increase the dose delivered to the kidneys. It would then be advisable to use cationic amino acid infusions to prevent these adverse effects. Another way to maximize the dose to the tumor while sparing healthy tissue is to administer radiolabeled antibodies locoregionally, or directly into the tumor cavity (153). This is the case of nimotuzumab radiolabeled with rhenium-188 in the management of high-grade gliomas in adults (154, 155). Indeed, some patients are not eligible for complete surgical resection or irradiation of lesions by conventional radiotherapy. Therefore, an uncontrolled, open-label, clinical phase I study was conducted to evaluate the safety and maximum tolerated dose of single intracavitary administration of radiolabeled nimotuzumab with ^188^Re, in 3 patients with anaplastic astrocytoma and 8 with glioblastoma multiforme. It is a humanized monoclonal antibody of IgG1 isotype that recognizes an epitope located in the extracellular domain of EGF-R receptors. Administration of a maximum activity of 10 mCi in brain tissue showed a high tumoricidal dose with acceptable irradiation of the kidneys, liver and bladder.

**Figure 5:**
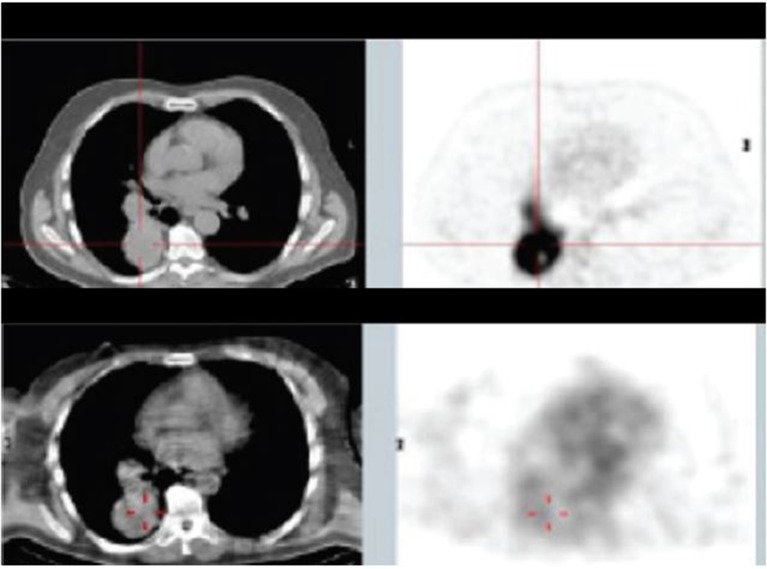
Patient from Phase Ia study with mediastinal and lung metastases: top panel—^18^FDG PET/CT 10 days before the study, lower panel—SPECT/CT of ^188^Re-6D2 mAb at 2 h after injection [from Klein et al. (156), available under the terms of the Creative Commons Attribution (CC BY)].

In consecutive Phase Ia and Phase Ib studies (156), Klein et al. demonstrated that ^188^Re-6D2, a radiolabeled IgM targeting melanin, was well tolerated, localized in melanoma metastases (Figure 5), and had antitumor activity, with a median overall survival of 13 months and no dose-limiting toxicities. The advantage of targeting melanin instead of ordinary antigens is that, in rapidly growing melanoma tumors, cell necrosis releases melanin into the extracellular space where it can easily be targeted (157). Moreover, melanin is insoluble, resistant to degradation, and can be expected to accumulate in targeted tissues.

Some other really intriguing potential applications of ^188^Re-labeled antibodies, but falling out of the scope of this review, have been proposed by Dadachova's team. They aim at treating infectious diseases, such as microbial or fungal infection (158, 159) or HIV (160).

### PRRT With ^188^Re

Peptides have several advantages over antibodies such as low immunogenicity, rapid penetration in the target tissue and clearance from plasma and non-target tissues. Moreover, due to the relatively short half-life of ^188^Re and the long circulating time of antibodies, radiolabeling peptides might be more suitable. Research on the labeling of peptides with ^188^Re has been very active, either on the search for the ideal chelating system (161) or on the quest for the analog having the highest affinity and stability (162, 163). A number of peptides have been radiolabeled with ^188^Re, mainly somatostatin derivatives (164–168). Other considered targets include gastrin releasing peptide receptor (GRPr) with bombesin (169) or GRPr-antagonist RM26 (170), α_V_β_3_ integrin (169), NK1 receptors, with Substance P (171), HCC with SP94 peptide (172), VEGFR (173) or GRP78, a specific cancer cell-surface marker (174). Much work has also been done on targeting melanoma, either through melanin or melanocortin-1 receptor (MC1-R) (162, 175, 176).

**Figure 6:**
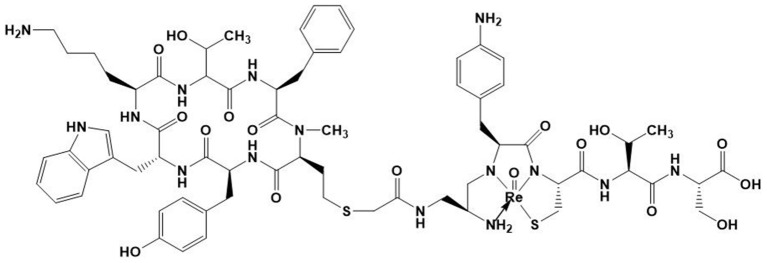
Structure of ^188^ReO-P2045.

There is, to date, however only one ^188^Re-labeled peptide that has been clinically investigated. It is ^188^Re-P2045 (Figure 6), an 11-amino acid peptide derived from ^99m^Tc-P829 (depreotide) targeting SST receptors, which has been studied in patients with advanced pulmonary cancer (177). 5 of the 8 patients had stabilized disease for at least 8 weeks, and median overall survival was 11.5 months. Nevertheless, this study has shown a dose delivered to the kidneys that can cause irreversible damage, which prevented further escalation. This renal toxicity can occur in the long term without having early indicators of this failure. Future challenges for the development of radiolabeled antibodies and peptides will notably be to minimize these toxicities, in particular to minimize renal failure.

## ^188^Re Particulates

### Radiolabeled Lipiodol and Microspheres for Liver Cancers

Primary and secondary liver tumors are a major cause of death, and their incidence is increasing. Among them, hepatocellular carcinoma (HCC), the major primary liver cancer, often appears on an underlying disease (fibrosis, cirrhosis) and is usually detected late, with a curative treatment which therefore can only be proposed to a small minority of patients. Taking advantage of the dual blood supply and rich vasculature of the liver, transarterial radioembolization (TARE) with radiolabeled Lipiodol or microspheres has demonstrated its interest for the management of HCCs at intermediate to advanced stages and intra-hepatic metastases (178–180). Notably, two ^90^Y-microspheres devices (SIR-Sphere® and TheraSphere®) have been successfully used for ~2 decades, and have been recently FDA-approved. Thanks to its on-site availability, and to its low-energy gamma-emission authorizing imaging, ^188^Re represents a potential alternative to ^90^Y.

#### Radiolabeled Lipiodol

**Figure 7:**
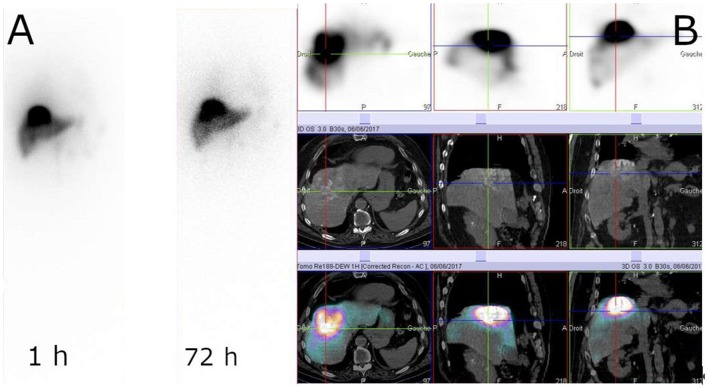
Example of ^188^Re-SSS biodistribution profile. Whole-body scintigraphy at 1 and 72 h **(A)** and SPECT/CT at 1 h **(B)** (Courtesy of Prof. Etienne Garin, Rennes, France).

There has been very active research on radiolabeling of Lipiodol with rhenium-188 (181). Three different ^188^Re-chelates are currently evaluated for the preparation of clinical ^188^Re-labeled Lipiodol, i.e., ^188^Re-HDD (182), ^188^ReN-DEDC (183) and ^188^Re-SSS (184), most clinical studies being carried out with the first one (185–198). ^188^Re-Lipiodol has been investigated in several early phase feasibility studies in non-operable HCC, with patients with advanced cirrhosis (189), or with extensive portal vein thrombosis (191), in second-line therapy to manage recurrences after a curative treatment (192, 193) and to stabilize patients on the liver transplant waiting list (190). To assess the maximum tolerated dose, several dose-escalation studies have been carried out (183, 186, 194, 199). The main at-risk organs are the lungs and healthy liver. In the frame of a Coordinated Research Project funded by the IAEA (200), Phase I (186) then Phase II (196) trials were undertaken in several countries. The overall results demonstrated favorable responses and potential usefulness of ^188^Re-Lipiodol for the therapy of HCC, which is now almost routinely used in several centers in India. One limitation of these studies is that, except the IAEA-sponsored trials, all of them included a very small number of patients, making it difficult to be conclusive. More trials, including larger cohorts of patients, are warranted. Another limitation, specifically with ^188^Re-HDD, is the low labeling yields and high urinary excretion (more than 40% at 72 h) (198). The next generation compounds, such as ^188^ReN-DEDC and ^188^Re-SSS, demonstrated higher yields and higher *in vivo* stabilities (183, 199) (Figure 7). A newly developed HDD complex (201) is expected to solve the problems encountered with the previous HDD, but no clinical data are available yet.

#### Radiolabeled Microspheres

**Figure 8:**
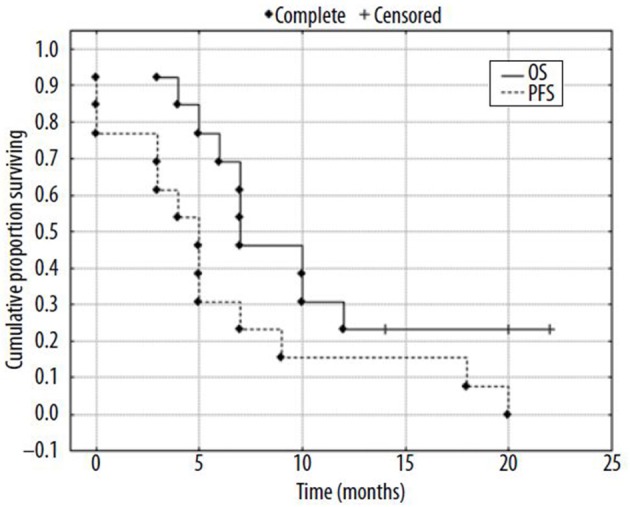
Kaplan-Mayer surviving curves for patients (*N* = 13) after radioembolization of liver tumors with ^188^Re-HSA microspheres [from Nowicki et al. (207), available under the terms of the Creative Commons Attribution Non Commercial-No Derivs 3.0 (CC BY-NC-ND 3.0)].

Different materials have been investigated for the preparation of ^188^Re-microspheres (202–205), but, to date, only human serum albumin (HSA) microspheres have made their way to the clinic. One advantage of HSA is that it is an approved drug, with ^99m^Tc-HSA routinely used in nuclear medicine centers. Two feasibility studies, with patients suffering from HCC or metastatic tumors from various origin, have been published (206, 207). Both studies demonstrated a high product stability, with a low urinary excretion (208), and good tolerance, with acceptable toxicity. In the first study, 2 patients out of 10 demonstrated a partial response (PR) at 3 months, while, in the second one, 5 out of 13 had a PR (Figure 8). These encouraging studies included a small number of patients, with heterogeneous tumors. Larger cohorts are mandatory to be able to conclude on the usefulness of this device.

### Radiocolloids and Liposomes

**Figure 9:**
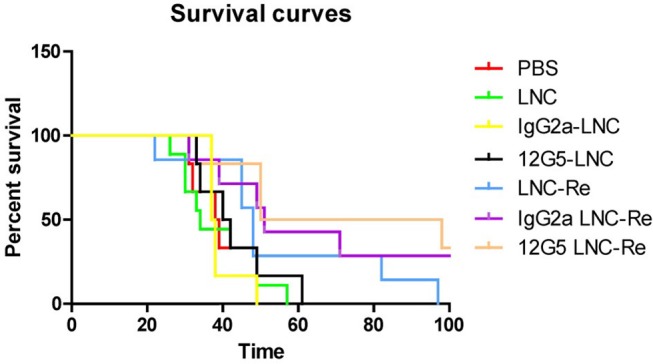
Kaplan Meier curves of mice treated with saline solution (PBS), blank LNC, immuno-LNCs (12G5-LNCs and IgG2a-LNCs) and internal radiation therapies (LNC^188^Re, IgG2a-LNC^188^Re, and 12G5-LNC^188^Re) after single infusion through convection-enhanced delivery into CXCR4-positive brain tumors [from Séhédic et al. (214), available under the terms of the Creative Commons Attribution Non Commercial 4.0 (CC BY-NC 4.0)].

An alternative route to target and deliver radioactivity into close contact with tumors that are spread out over the serous membrane of cavities and to tumor cells present in the malignant effusions, is to inject the radiopharmaceutical directly into these cavities, as exemplified above with RIT. Intracavitary radionuclide therapy can be applied to the pleural, pericardial and peritoneal cavities, intrathecally and also into cystic tumors. For this purpose, radiolabeled colloids have been proven safe and effective (209), but most of the research conducted with ^188^Re has been preclinical. Melanoma-bearing mice have been treated with intra-peritoneal injection of ^188^Re-colloids, leading to an increased survival of the treated animals compared to control group (210). ^188^Re-microspheres embedded in a fibrin glue gel have been proposed as a potential adjuvant treatment to be applied in the tumor bed immediately after resection of glioblastomas (211). ^188^Re-loaded lipid nanocapsules demonstrated outstanding efficacy in rat glioblastoma models, after convection-enhanced delivery into the tumor, with a significant increase in the survival and induction of an immune response (212–214) (Figure 9). A Phase I/II study is expected to start soon. A radiobiological study by Hrycushko et al. aimed at demonstrating the potential usefulness of ^188^Re-loaded liposomes to prevent recurrence after surgical resection of breast tumors. Based on biodistribution results in rats, dose distributions were modeled and radiobiological indexes determined, following direct injection of ^188^Re-liposomes into the lumpectomy cavity (215, 216). The same group also carried out a similar work with head and neck squamous cell carcinoma, following direct intratumoral infusion of ^99m^Tc-labeled liposomes (217, 218). These theoretical results would need to be confirmed *in vivo*. There is currently a clinical trial running in Taiwan, on ^188^Re-liposomes in patients with primary solid tumor in advanced or metastatic stage (NCT02271516). To date, only preliminary results have been published (219). One patient with advanced serous ovarian adenocarcinoma and one patient with endometrioid ovarian adenocarcinoma were treated twice with intraperitoneal injection of ^188^Re-BMEDA-liposome, leading to a decrease of cancer antigen 125 in serum, used as a biomarker of treatment response, and a longer than expected survival. The completion of the trial is thus expected to confirm these results.

Another intracavitary application of ^188^Re and other β-emitter-labeled radiocolloids is the radionuclide treatment of benign diseases by intra-articular injection in cases of persistent synovial effusions due to rheumatoid arthritis and other inflammatory joint disease (220).

### Brachytherapy of Skin Cancers

A particularly original and attractive treatment modality using ^188^Re-particulates is the use of ^188^Re-colloids within a brachytherapy device for skin cancer treatment. Radioactive patches made of nitrocellulose filter paper loaded with ^188^Re-tin colloids were developed by Jeong et al. (221). This method was successfully used in patients with keloids, a benign dermal fibro proliferative tumor, and non-melanoma skin cancers (222, 223).

**Figure 10:**
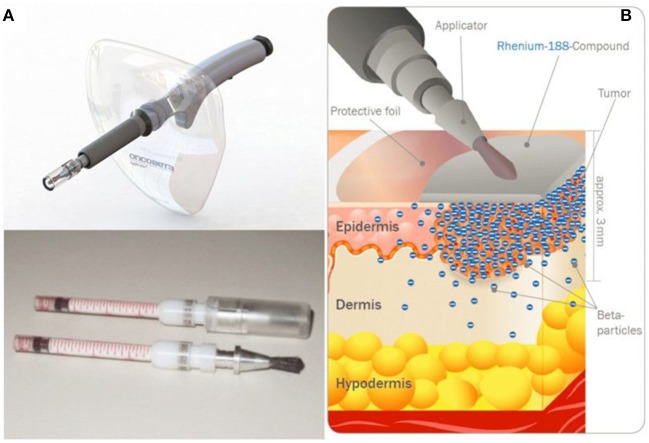
Rhenium-SCT device developed by OncoBeta^®^ GmbH (Garching, Germany). Applicator and dispensing carpoules filled with ^188^Re-cream **(A)** and illustration of the principle **(B)** (Courtesy of Dr. Shannon Brown III, OncoBeta®).

An alternative device embeds radiocolloids inside a mix of synthetic acrylic co-polymers inert matrix, and tensioactives, and has been investigated in patients with basal and squamous cell carcinomas (224). Fifty-three patients with histologically confirmed basal cell carcinoma (BCC) or squamous cell carcinoma (SCC) were treated. Three months later, complete healing was obtained in 100% of the treated patients; even after a single application in 82% of the cases. After a mean follow-up of 51 months, no clinical relapses were observed in the treated patients, and histological examination confirmed complete tumor regression. The inert matrix containing the ^188^Re is able to adapt to every skin surface without contamination, imparting an accurate distribution of dose and sparing the healthy tissue. The technology was further improved, and, in a more recent study (225), 29 BCC and 14 SCC patients were treated. One patient was lost to follow-up before wound closing, but wound healing was complete for all other 42 patients (average 65 days), with no side effects to be reported. During the period of follow-up (average 288 days), no single recurrence occurred. This ^188^Re-cream can be deposited through a CE-labeled applicator (Figure 10), now commercially available under tradename Rhenium-SCT® (Skin Cancer Therapy), from OncoBeta® GmbH (Garching, Germany) and this system is routinely used in Italy and South-Africa, where it is an approved therapy for the treatment of BCC and SCC, including Bowen's disease, in patients with comorbidities, when surgical intervention is not possible or conventional therapies cannot be expected to provide a satisfactory cosmetic result due to the anatomical location. This treatment modality is particularly interesting when surgery is not desirable, as in the case of SCC of the penis (226). In that study, 15 patients, ranging in age from 31 to 92 years, were treated with the Re-SCT® brachytherapy kit. After one to seven different previous treatments (for multifocal lesions), 12 patients were in complete remission, 2 did not respond, and one patient was lost to follow-up, with a mean follow-up of 51 months. Most importantly, this technique was painless and spared the anatomical integrity of the organ. In addition to BCC and SCC, this method was investigated in patients suffering from extramammary Paget's disease (EMPD) (227). Five patients with primary or secondary EMPD were successfully treated, in one or two sessions, with a mean follow-up of 34 months. All patients showed complete remission at the end of the treatments. Four patients later had relapse, either inside or at the periphery of the treated area.

## Conclusion

Many clinical trials, from feasibility studies to Phase II studies, have been carried out with Rhenium-188-labeled radiopharmaceuticals and have demonstrated the feasibility and clinical usefulness of ^188^Re-labeled radiopharmaceuticals for a wide range of pathologies, especially in oncology, but also for benign diseases. Despite the advent of more “user-friendly” radionuclides such as ^90^Y and ^177^Lu, ^188^Re still holds great promise with compounds like ^188^Re-HEDP for bone pain palliation or ^188^Re-Lipiodol for liver cancers. Large cohorts of patients are now needed for these agents to find their place within a very competitive environment, with therapies already in use. Brachytherapy of skin cancers also appears particularly attractive, with no direct concurrent for these pathologies. Besides, the development of new ^188^Re radiotracers, with novel, more stable, cores like tricarbonyl, HYNIC, or nitrido, should lead to molecules with more favorable pharmacokinetic characteristics. More widespread use of ^188^Re-radiopharmaceuticals will now rely on availability of fully pharmaceutical grade generators and wide clinical proofs of its interest in radionuclide therapy, particularly with the possibility of having a matched theranostic pair with ^99m^Tc.

## Author Contributions

All authors listed have made a substantial, direct and intellectual contribution to the work, and approved it for publication.

### Conflict of Interest Statement

The authors declare that the research was conducted in the absence of any commercial or financial relationships that could be construed as a potential conflict of interest.
